# Long-term changes in serum silver concentrations after extremity reconstruction with silver-coated megaprostheses

**DOI:** 10.1038/s41598-022-16707-0

**Published:** 2022-07-29

**Authors:** Maria Anna Smolle, Marko Bergovec, Susanne Scheipl, Walter Gössler, Florian Amerstorfer, Mathias Glehr, Andreas Leithner, Jörg Friesenbichler

**Affiliations:** 1 Department of Orthopaedics and Trauma, Medical University of Graz, Auenbruggerplatz 5, 8036 Graz, Austria; 2 Institute of Analytic Chemistry, Karl-Franzens University, Graz, Austria

**Keywords:** Outcomes research, Orthopaedics

## Abstract

Silver-coated megaprostheses are considered to reduce infection rate following reconstruction of bone defects in tumour surgery or revision arthroplasty. However, little is known about systemic silver exposure and possible side effects. The aim of this study was to analyse serum silver concentrations in patients with silver-coated megaprostheses over a prolonged time period. Between 2004 and 2016, 46 patients (52.2% female, mean age at surgery 47.1 ± 24.2 years) received silver-coated megaprostheses for septic (n = 26) or oncological (n = 17; main implant since 2013) indications, or aseptic loosening (n = 3). Blood was drawn from all patients within the first few days following surgery (without silver ion levels) and thereafter every 6 months at the outpatient department (with silver ion levels). Inductively coupled plasma mass spectrometry was used to determine silver ion levels. Median follow-up was 47.3 months (IQR: 16.1–78.9). Overall, 29 revision surgeries became necessary in 20 patients, equivalent to a cumulative complication rate of 63.0%. Revisions were most commonly for periprosthetic joint infections (PJIs, n = 12) and instability/soft tissue problems (n = 10). Revision-free implant survival was 81.4%, 42.3% and 35.2% at one, 5 and 10 years. Incidence of local argyria was 8.7% (n = 4). Silver ion levels at two or more consecutive time points during follow-up were available for 26 patients. An increment of silver levels within the first months (“run-in”) was observed, followed by an unspecific undulating course. Median initial and latest follow-up (median, 49.5 months) serum silver ion levels were 16.0 ppb (IQR: 9.1–29.1) and 7.4 ppb (IQR: 2.7–14.1), respectively. According to the multivariate mixed linear random-effects model, development of PJI was associated with significantly higher silver ion levels over time (p = 0.002), irrespective of time from surgery (p = 0.274). In the current series, a cumulative complication rate of 63.0% was observed for patients receiving silver-coated megaprostheses for septic of oncological indications. An overall unspecific course of silver ion concentration was present. Development of PJI was significantly associated with increased silver ion levels over time. Yet, no systemic complication associated to high silver levels occurred. It can be concluded that silver-coated implants constitute a safe solution for megaprosthetic reconstruction, but monitoring of silver concentrations is recommended.

## Introduction

Modular megaprostheses are usually used for extremity reconstruction in case of limb salvage surgery following resection of malignant soft tissue or bone tumours, as well as in case of massive bone loss in the setting of revision arthroplasty^1^. In the beginnings of revision surgery and orthopaedic oncology, most implants available were custom made and very expensive. With time, however, several modular “of the shelf” devices have become available on the market. In the literature, different complication and implant survival rates are reported after megaprosthetic reconstruction in septic revision surgery or orthopaedic oncology, with the procedure itself known as complication-prone^1–8^. Thus, whilst the rate of limb sparing surgery has steadily increased, high infection rates (up to 50%) are one of the most common complications resulting in secondary amputation, if uncontrolled^1–3,6,7,9–13^.

Treatment of periprosthetic joint infections (PJI) range from DAIR (debriment, antibiotics and implant retention) to one-stage procedures for early infections and one-stage or two-stage procedures for late infections, accompanied by antibiogram-adapted antibiotic regimens. Yet, secondary amputation may needed as ultimate treatment in some patients^1,7,11,14^.

Various antimicrobial coatings on the surface of endoprosthetic devices have been tested, attempting to reduce PJIs rates, whereupon silver coating showed the best results^1,6,7,12^. It has been applied as surface coating, as small flat plates or as powder. Nowadays, silver molecules are incorporated in several medical devices like catheters, grafts, suture materials and endoprostheses^6^. Silver coating enables the continuous release of silver ions from the implants’ surface by dissolution, thus providing an antibacterial environment. However, after long term use, silver molecules can accumulate in various tissues including skin, cornea, mucous membranes, upper abdominal organs and nails, leading to the so called silver disease^4,5,9,15^. Furthermore, known adverse side effects of high silver ion levels are neurological symptoms^16^ and local argyria, although Glehr et al*.*^9^ reported no direct correlation between local discoloration and systemic silver concentrations. Several studies have reported on outcome and implant survival rate of silver-coated megaprostheses but little is known about systemic silver exposure and long-term effects^1–8,17^. The aim of this study was to report longitudinal blood silver ion concentration changes in patients undergoing megaprosthetic reconstruction with silver-coated implants. Furthermore, complications, revision-free survival rates and factors associated with altered silver ion levels over time, were assessed.

## Materials and methods

For this retrospective study, 46 patients (24 females [52.2%], mean age 47.1 ± 24.2 years) treated between 2004 and 2016 with modular megaprostheses for septic indications (n = 26), oncological reconstructions (n = 17), or aseptic loosening (n = 3) were included. Notably, 9 out of the 26 patients treated for septic indications had previously received an endoprosthetic reconstruction for tumour surgery. In all but 4 cases, the modular megaprosthetic device MUTARS (*Implantcast GmbH, Buxtehude, Germany*) with a galvanised silver coating, was used for reconstruction. In the remaining 4 cases, a silver-coated arthrodesis implant by the same manufacturer had been used. Additionally, 5 selected oncological cases had been treated with silver-coated megaprostheses from 2007 onwards. Since 2013, silver-coated megaprostheses have become the main reconstruction system at the authors’ institution. Median postoperative follow-up was 47.3 months [interquartile range: 16.1–78.9 months]. At latest follow-up, 31 patients were alive (67.4%) and 11 had died (23.9%), whilst 4 patients were lost to follow-up (8.7%). According to the manufacturer (*Implantcast, Buxtehude, Germany*), thickness of the silver coating on each modular component was 20 μm, containing 0.172 μg silver particles per square centimeters. This composition had not changed over the inclusion period. Thus, the amount of silver implanted was calculated by the length of resection multiplied with the components’ silver content.

Determination of serum silver concentration was done within several days post-surgery, and subsequently every 6 months at the outpatient department. Serum silver concentration measurement was done using inductively coupled plasma mass spectrometry (*ICP-MS, Agilent 7500ce; Agilent, Waldbronn, Germany*) after microwave-assisted digestion with nitric acid in a microwave-heated autoclave (*MLS ultraClave III; MLS-Mikrowellenlaborsysteme, Leutkirch, Germany*). The exact method has been described in the series by Glehr et al.^9^.

Liver and kidney parameters were obtained in light of potential organ failure upon systemic accumulation of silver ions. Signs of systemic silver-associated side effects and local argyria were evaluated in all patients upon routine postoperative clinical check-ups by means of thorough history taking and physical examination. In case of local argyria, neurological assessment was performed, as previously described^9^.

Notably, baseline silver ion concentrations were available in 33, and at least two measurements in 26 patients of the entire cohort. Statistical analyses regarding changes in silver ion concentrations were thus performed including 26 patients only. Of these, 25 had a lower-limb megaprosthesis, and one a proximal humeral megaprosthesis. None of the patients with a knee arthrodesis implant had more than one measurement of silver ion levels available.

Implant-associated complications were classified into five categories, as proposed by Henderson et al.^18,19^. The diagnosis of PJI was provided on the basis of international consensus for the diagnosis of periprosthetic infection^20^. As reinfection, we defined a clinically and microbiologically recurrence of local PJI after an antibiotic-free period and the absence of clinical symptoms for at least 6 weeks.

### Statistical analysis

Statistical analyses were performed with Stata Version 16.1 for Mac (StataCorp, College Station, TX, USA). Means (normally distributed variables) and medians (non-normally distributed variables) were provided with corresponding standard deviations (SDs) and IQRs, respectively. Kaplan–Meier method and log-rank test was performed to assess revision-free survival (any Henderson type), mechanical complication-free survival (Henderson types I, II and III), and infection-free survival (Henderson type IV) up to 10 years postoperatively, with any complication, mechanical complications, and periprosthetic joint infections, as the respective endpoints. Wilcoxon matched pair signed rank test was used to assess differences between non-normally distributed continuous paired variables. Spearman’s rank correlation coefficient (rho) was performed to assess correlations between non-normally distributed continuous variables. The assumption that silver ion level measurements over time were missing completely at random (MCAR) was verified with Little’s X^2^ test (Stata command mcartest; p = 0.311)^21^. Thus, univariate and multivariate linear random-effects models could be performed to assess correlations between longitudinal changes of silver ion level measurements and demographic as well as clinical variables. A p-value of < 0.05 was considered statistically significant.

The study was performed in accordance with the Declaration of Helsinki, has been approved by the local institutional review board (Ethics committee of the Medical University of Graz, Austria; EK-Nr. 21-131 ex 09/10), and all patients gave their informed consent.

## Results

Sixteen proximal femoral, 6 distal femoral, 6 total femoral, and 3 intercalary femoral reconstructions were performed. Furthermore, 6 and 5 reconstructions were located at the proximal tibia and humerus, respectively. In 4 additional cases, a sliver-coated arthrodesis implant was used for knee arthrodesis. Mean osseous resection and reconstruction length was 17.8 ± 11.4 cm. Mean amount of silver implanted per patient was 3.1 ± 2.0 μg. At latest follow-up of 47.3 months (IQR: 16.1–78.9), 12 out of 26 patients with silver-coated megaprostheses for septic revision were alive without evidence of infection, 5 alive without evidence of disease (oncological patients), 7 had died of unknown causes, and two had been lost to follow-up. All three patients with mechanical wear as the indication for implantation of a silver-coated megaprosthesis were alive without any problems. Of the 17 patients with underlying malignancy as the reason for implantation of the silver-coated megaprosthesis 10 were alive without disease, one was alive with disease, 4 had died of disease, and two were lost to follow-up. Median creatinine clearance of the 46 patients at time of implantation was 0.75 mg/dl (IQR: 0.61–0.88). At the same time, the hepatic parameters (gamma-GT, ALT and AST), indicative of liver function were also within the normal limits, except for nine patients having either received preoperative chemotherapy (n = 3) or high dose antibiotics for PJI with toxicological side effects on the liver (n = 6). Further demographic data is shown in Table 1.

**Table 1:** Descriptive analysis of the entire patient cohort.

	(n; %)
**Gender**	
Males	22 (47.8%)
Females	24 (52.2%)
**Indications**	
Primary surgery	20 (43.5%)
Revision surgery	26 (56.5%)
**Type of reconstruction**	
Proximal femur	16 (34.8%)
Distal femur	6 (13.0%)
Total femur	6 (13.0%)
Proximal tibia	6 (13.0%)
Proximal humerus	5 (10.9%)
Arthrodesis implant	4 (8.7%)
Intercalary femur	3 (6.5%)
**Age at surgery (in years; mean ± SD)**	47.1 ± 24.2
**Reconstruction length (in cm; mean ± SD)**	17.8 ± 11.4
**Amount of silver in coating (in μg; mean ± SD)**	3.1 ± 2.0
**Creatinine at time of implant (in mg/dl; median, IQR)**	0.75 [0.61–0.88]
**GGT at time of implant (in U/l; median, IQR)**	26.0 [16.0–49.0]
**ALT at time of implant (in U/l; median, IQR)**	20.0 [12.0–30.0]
**AST at time of implant (in U/l; median, IQR)**	20.0 [17.0–29.0]
**Time of follow-up (in months; median, IQR)**	47.3 [16.1–78.9]

At a median latest follow-up of 31.0 months (IQR: 6.2–58.2), kidney and hepatic enzyme levels were available in 37 patients. Median creatinine clearance was 0.73 mg/dl (IQR: 0.59–0.82; n = 37), median ALT 18.0 U/l (IQR: 12–23; n = 37), median GGT 25.5 U/l (IQR: 14.5–57.5; n = 36), and median AST 22.0 U/l (IQR: 18.0–31.5; n = 36).

Wilcoxon matched pair signed rank test showed no significant difference between laboratory parameters at time of implantation and latest follow-up (creatinine clearance: p = 0.368; ALT: p = 0.095; GGT: p = 0.280; AST: p = 0.416).

### Complications

**Figure 1:**
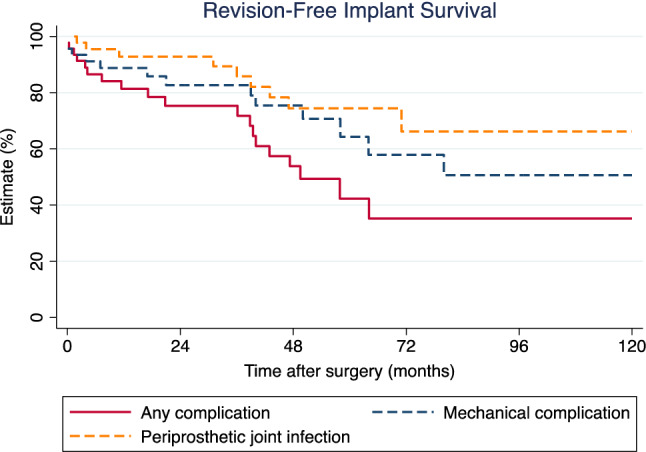
Kaplan–Meier curve for overall implant revision-free implant survival (any Henderson type; solid red line), mechanical complication-free survival (Henderson types I, II, III; dashed blue line), and periprosthetic joint infection-free survival (Henderson type IV; dashed orange line).

Within the entire cohort, there were 29 revision surgeries for implant-associated complications in 20 patients (14 with previous failed arthroplasty), of whom 6 required more than on one revision. This amounted to a cumulative complication rate of 63.0%. According to the Henderson classification^18,19^, there were 10 type I failures (instability and soft tissue problems; 34.5%), 4 type II failures (aseptic loosening, 13.8%), 2 type III failures (periprosthetic fractures, 6.9%), 12 type IV failures (infections, 41.4%) and one type V failure (relapse of tumour, 3.4%). The calculated implant survival for any complication needing revision was 81.4%, 42.3% and 35.2% at one, 5 and 10 years following primary implantation of the silver coated megaprosthesis, respectively (Fig. 1).

### Mechanical failures

Overall, 16 mechanical complications occurred in 13 patients, including 5 luxations (one of which was a luxation-elongation), 4 aseptic loosenings, 2 periprosthetic fractures, one disconnection of modular components, one haematoma formation, one perforation, one aseptic early postoperative wound dehiscence, and one patellar impingement.

The calculated implant survival for mechanical failures needing revision was 88.7% at one year, 64.3% at 5 years, and 50.6% at 10 years following implantation (Fig. 1).

### Infections

There were 12 cases of PJI necessitating revision surgery in 10 patients (two developed a second PJI after successful treatment of the first), resulting in a cumulative (re)-infection rate of 26.1%. Of these 10 patients, nine had received the silver-coated megaprosthesis following previous PJI. The tenth patient developed PJI 4 months after wide resection for osteosarcoma and reconstruction with a silver-coated total humerus implant. In patients having received the silver-coated megaprosthesis upon septic revision surgery, median time from implantation to development of another PJI was 47.9 months (IQR: 18.9–51.6 months). Microorganisms causing PJI included Staphylococcus epidermidis (n = 5), Enterococcus faecalis (n = 3), Staphylococcus aureus (n = 3), and Streptococcus agalactiae (n = 1). All patients with PJI were treated with a two-stage procedure as therapy of choice, secondary amputation became ultimately necessary in two patients. The calculated PJI-free survival was 92.8% at 1 year, 74.4% at 5 years, and 66.1% at ten years of follow-up (Fig. 1).

### Argyria

**Figure 2:**
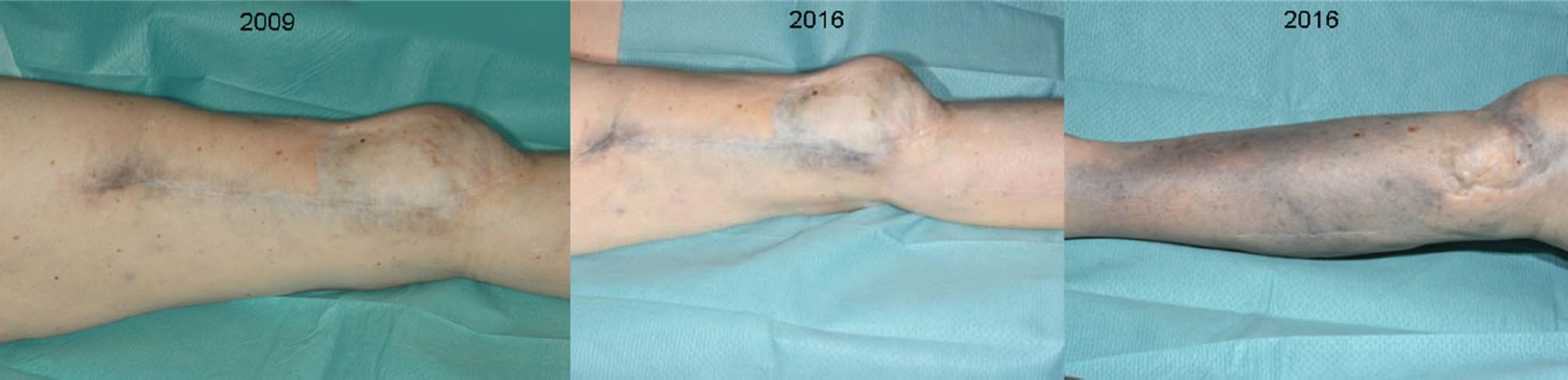
Argyria after distal femoral reconstruction with progressive discoloration during a follow-up period of 7 years (2009, left; 2016, mid and right).

Four out of 46 patients developed idiosyncratic local argyria, resulting in an overall incidence of 8.7%. There was no difference between patients with local argyria and those without regarding implant size (p = 0.348), initial systemic silver ion concentrations (p = 0.377) or those latest follow-up (p = 0.696; for 26 patients with ≥ measurements), as already discovered by Glehr et al*.*^9^ in a study including some of the patients herein analysed. Although local argyria may just constitute a cosmetic problem, we observed pronounced progression of skin discoloration in one patient, despite strict avoidance of any direct sun exposure (Fig. 2).

### Serum silver concentrations

Baseline silver ion concentrations had been obtained in 33 patients, amounting to a median of 16.0 [IQR: 9.1–29.1] ppb in the early postoperative period. In 26 of these, at least two serum silver ion measurements over time were available, allowing for longitudinal assessment of silver ion level change. The latest median silver ion levels in the blood stream, obtained at 49.5 months (IQR: 38.0–92.0 months), were 7.4 ppb (IQR: 2.7–14.1). There was no significant correlation between silver ion concentrations at latest follow-up and most recent creatinine clearance (Spearman’s rho: 0.226; p = 0.299), ALT (Spearman’s rho: 0.212; p = 0.331), AST (Spearman’s rho: -0.100; p = 0.649), or GGT levels (Spearman’s rho: 0.221; p = 0.311).

**Figure 3:**
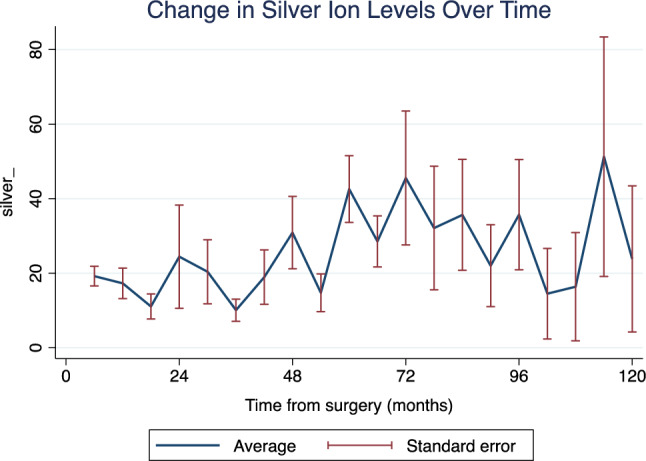
Distribution of silver ion levels over time.

During follow-up, we observed an increment of systemic silver concentrations in all 26 patients, followed by an unspecific undulating course (Fig. 3).

Univariate linear random effects model revealed no statistically significant correlation between time from surgery and longitudinal change in silver ion levels (p = 0.091; Table 2).

**Table 2:** Univariate linear random effects model for change in silver ion levels over time (n = 26).

Silver ion levels	Coefficient	Standard error	95% confidence interval	p-value
Time from surgery (per 6 months)	0.11	0.06	− 0.02; 0.23	0.091
Age at surgery (per 1 year increase)	0.11	0.18	− 0.24; 0.47	0.525
Female Gender	3.87	8.13	− 12.07; 19.81	0.634
Mechanical Revision	− 10.19	12.35	− 34.40; 14.01	0.409
Periprosthetic joint infection	25.0	7.62	10.07; 39.9	**0.001**
Argyria	12.81	10.43	− 7.64; 33.27	0.220

Significant values are given in bold.

**Figure 4:**
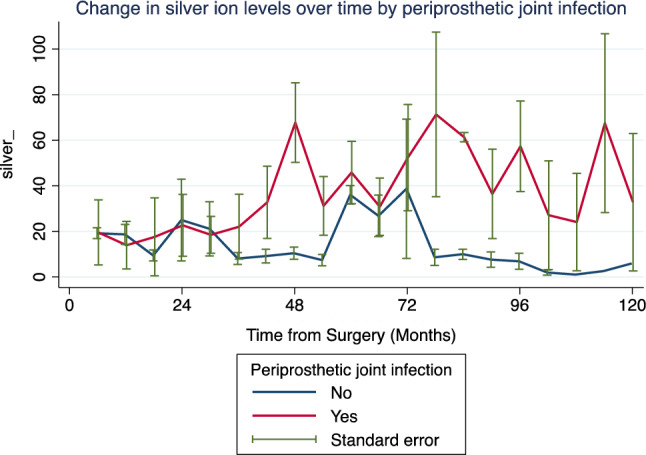
Change in silver ion levels over time, divided by patients without (blue line) and with (red line) periprosthetic joint infection.

Likewise, age at surgery (p = 0.525), female gender (p = 0.634), local argyria (p = 0.220) and mechanical revision (p = 0.409) were not significantly correlated with changes in silver ion levels over time. Notably, development of periprosthetic joint infection was significantly associated with increased silver ion levels over time (p = 0.001; Table 2, Fig. 4).

In the multivariate linear random-effects model, development of periprosthetic joint infection remained significantly associated with increase in silver ion levels over time (p = 0.002), irrespective of time from surgery (p = 0.274; Table 3).

**Table 3:** Multivariate linear random-effects model for longitudinal change in silver ions (n = 26).

Silver ion levels	Coefficient	Standard error	95% confidence interval	p-value
Time from surgery (per 6 months)	0.06	0.05	− 0.05; 0.17	0.274
Periprosthetic joint infection	24.43	7.74	9.26; 39.60	**0.002**
Constant	11.64	2.43	20.85	**0.013**

Significant values are given in bold.

## Discussion

In the current study, a cumulative complication rate of 63.0% was observed in a high-risk population of patients having undergone surgery with silver-coated megaprostheses. Silver ion levels initially increased in all patients, followed by an undulating course. Whist factors as patient age and time from surgery were not significantly associated with changes in silver ion levels over time, development of PJI showed a significant association with increasing silver ion levels. Four patients developed local argyria. No systemic complications attributable to high silver ion levels could be observed.

Shivaram et al.^22^ investigated the long-term silver release from the surface of porous coated titanium implants. Comparable to the current series, they observed an initial burst release due to leaching of silver ions with gradual stabilization over time. The stabilization times varied depending on factors such as experimental conditions, particle size, concentration, pH gradient and aggregation kinetics. Notably, a faster release of silver ions was observed in presence of low pH, also often present in case of joint infection^22,23^.

In 2007, Hardes et al*.*^3^ reported on silver ion levels in 20 patients within the first 24 months after implantation of a silver coated MUTARS device. Their results were comparable to the results of the current series without any systemic complications associated to the silver coating. Thus, Hardes et al.^3^ stated that silver-coated megaprostheses might be a sufficient tool for prevention of PJI in tumor and revision surgery.

Silver ion levels after implantation of the modified Mega System C with PorArg^®^ silver coating (Link GmbH, Hamburg, Germany) in 33 patients were reported by Scoccianti et al.^24^. They discovered mean silver ion concentrations in blood of 2.01 ppb at 36 months following primary surgery^24^. Furthermore, neither argyria nor any other local or systemic side effects related to silver were observed^24^. Our data and the one from Hardes et al*.*^3^ demonstrate that clinically significant levels of silver ions are still measurable in blood and urine for as long as two (Hardes’ series), and up to a median of 4.1 years (current series) following surgery. Therefore, a long-lasting local active effect against bacteria colonisation can be supposed, without serious systemic side effects. Yet, a possible local adverse effect of silver is argyria, with the skin becoming blue or bluish-grey. It is usually localised, but may also occur diffusely. Data about argyria in patients bearing silver-coated megaprostheses are inhomogeneous, with a reported incidence ranging from 0% to as high as 23%^9^. In the current series of 46 patients, four cases (8.7% of patients) of idiosyncratic local argyria were observed. Glehr et al. analysed parts of the same cohort in 2013, and discovered no correlation between systemic silver concentrations and implant size, or differences in silver concentrations between affected and non-affected patients^9^. Although predominantly constituting a cosmetic problem, we observed am enormous progression of skin discoloration with time.

Silver coatings are recommended by several authors especially in revision surgery or patients at-risk for PJI^1,6,7,11,12,25^. In our cohort of at-risk patients receiving silver-coated megaprostheses for orthopaedic oncology, septic revision, as well as aseptic loosening, a PJI rate of 26.1% was observed, being at the upper limit reported in literature^1,2,6,7,9–11,13^. Notably, of 10 patients with PJI during follow-up, all but one developed it following implantation of the silver-coated megaprosthesis for septic revision surgery. In this context, significantly higher silver ion levels over time were observed in case patients developed PJI, wherefore close monitoring of silver ion levels especially in these patients is recommended.

According to an animal study by Gosheger et al.^2^, the type of alloy also seems to have an influence on development of PJI, with significantly higher PJI rates found in implants made of Co-Cr alloy compared to titanium (31% vs. 14%). They also reported a significant reduction of infection rate from 47 to 7% when using silver coated devices compared to titanium devices^8^. This finding was supported by the results of Donati et al*.*^12^, showing a marked reduction of early and late infections in silver coated prostheses (7.9%) compared to titanium devices (16.7%). In another study, Hardes et al*.*^25^ also reported on a reduction of PJIs from nearly 18% in uncoated titanium prostheses to 6% when using silver coated implants over a period of 5 years. Wafa et al*.*^6^ likewise discovered lower PJI rates in patients with silver-coated endoprostheses compared to controls (12% vs. 22%). In their cohort, 70.6% of patients had received a silver-coated implant for septic revision surgery, explaining the higher overall (re)-infection rate^6^. Streitbuerger et al*.*^7^ reported on an overall infection rate of 11% for silver coated proximal femoral MUTARS megaprostheses for proximal femoral reconstruction in tumour surgery. The calculated event free survival was 90% at 5 and 10 years of follow-up, which is better than the 35.2% observed in the recent series during the same period. Recently, a systematic review and meta-analysis by Fiore et al. reported on an overall infection rate of 9.2% in silver coated megaprostheses compared with 11.2% upon use of uncoated devices^17^. Furthermore, the authors observed significantly lower re-infection rates in case the silver-coated implants had been used upon revision surgery (13.7%) in comparison to uncoated devices (29.2%)^17^. Thus, they reached the conclusion that silver-coated endoprostheses can effectively reduce risk for another PJI in patients after two-stage septic revision surgery^17^. However, most studies available for inclusion were developer publications and potentially biased due to treatment assignment or patient selection^17^.

### Limitations

There are several limitations of the current study: first, the number of patients included is small, with varying indications for the use of silver-coated megaprostheses, eventually limiting the interpretation of complication- and PJI-rates observed due to heterogeneity. Second, discontinuous follow-up of patients was a limiting factor, with long-term silver ion levels available in 26 out of 46 patients only. This may have biased the results obtained. Third, confounding factors as use of another silver coated implant upon PJI were not considered in the current analysis. Fourth, it could not be clarified whether differences in silver ion concentrations are present between megaprostheses in non-weight-bearing and weight-bearing areas. On the other hand, advantages of the study include the fact that only one implant system was used over many years, and silver ion levels up to a median of 49.5 months were available.

## Conclusion

To the best of the authors’ knowledge, this is the first series reporting on long-term silver ion levels in patients treated with silver-coated megaprostheses. Silver may not be the ‘final solution' to prevent development PJI in megaprosthetic reconstructions, but it may well constitute a useful weapon in patients at risk for PJI due to previous septic complications, or orthopaedic oncological surgery. Based on the herein observed silver ion levels with time, corroborating previous findings, silver-coated devices seem to be safe without serious side effects. However, continuous monitoring of silver concentrations is still recommended, especially in case patients have developed PJI. Further long-term studies will be necessary to prove the effectiveness and secureness of silver coatings.

## Data Availability

The datasets used and/or analysed during the current study available from the corresponding author on reasonable request.

## References

[1] Zajonz D, Birke U, Ghanem M, Prietzel T, Josten C, Roth A, Fakler JKM (2017). Silver-coated modular Megaendoprostheses in salvage revision arthroplasty after periimplant infection with extensive bone loss—a pilot study of 34 patients. BMC Musculoskelet. Disord..

[2] Gosheger G, Goetze C, Hardes J, Joosten U, Winkelmann W, von Eiff C (2008). The influence of the alloy of megaprostheses on infection rate. J. Arthroplasty.

[3] Hardes J, Ahrens H, Gebert C, Streitbuerger A, Buerger H, Erren M, Gunsel A, Wedemeyer C, Saxler G, Winkelmann W (2007). Lack of toxicological side-effects in silver-coated megaprostheses in humans. Biomaterials.

[4] Sudmann E, Vik H, Rait M, Todnem K, Andersen KJ, Julsham K, Flesland O, Rungby J (1994). Systemic and local silver accumulation after total hip replacement using silver-impregnated bone cement. Med. Prog. Technol..

[5] Trop M, Novak M, Rodl S, Hellbom B, Kroell W, Goessler W (2006). Silver-coated dressing acticoat caused raised liver enzymes and argyria-like symptoms in burn patient. J. Trauma.

[6] Wafa H, Grimer RJ, Reddy K, Jeys L, Abudu A, Carter SR, Tillman RM (2015). Retrospective evaluation of the incidence of early periprosthetic infection with silver-treated endoprostheses in high-risk patients: Case-control study. Bone Joint J..

[7] Streitbuerger A, Henrichs MP, Hauschild G, Nottrott M, Guder W, Hardes J (2019). Silver-coated megaprostheses in the proximal femur in patients with sarcoma. Eur. J. Orthop. Surg. Traumatol..

[8] Gosheger G, Hardes J, Ahrens H, Streitburger A, Buerger H, Erren M, Gunsel A, Kemper FH, Winkelmann W, Von Eiff C (2004). Silver-coated megaendoprostheses in a rabbit model—An analysis of the infection rate and toxicological side effects. Biomaterials.

[9] Glehr M, Leithner A, Friesenbichler J, Goessler W, Avian A, Andreou D, Maurer-Ertl W, Windhager R, Tunn PU (2013). Argyria following the use of silver-coated megaprostheses: No association between the development of local argyria and elevated silver levels. Bone Joint J..

[10] Malawer MM, Chou LB (1995). Prosthetic survival and clinical results with use of large-segment replacements in the treatment of high-grade bone sarcomas. J. Bone Joint Surg. Am..

[11] Schmidt-Braekling T, Streitbuerger A, Gosheger G, Boettner F, Nottrott M, Ahrens H, Dieckmann R, Guder W, Andreou D, Hauschild G (2017). Silver-coated megaprostheses: Review of the literature. Eur. J. Orthop. Surg. Traumatol..

[12] Donati F, Di Giacomo G, D'Adamio S, Ziranu A, Careri S, Rosa M, Maccauro G (2016). Silver-coated hip megaprosthesis in oncological limb savage surgery. Biomed. Res. Int..

[13] Zajonz D, Zieme A, Prietzel T, Moche M, Tiepoldt S, Roth A, Josten C, von Salis-Soglio GF, Heyde CE, Ghanem M (2016). Periprosthetic joint infections in modular endoprostheses of the lower extremities: A retrospective observational study in 101 patients. Patient Saf. Surg..

[14] Jacobs AME, Valkering LJJ, Benard M, Meis JF, Goosen JHM (2019). Evaluation one year after DAIR treatment in 91 suspected early prosthetic joint infections in primary knee and hip arthroplasty. J. Bone Jt. Infect..

[15] Tomi NS, Kranke B, Aberer W (2004). A silver man. Lancet.

[16] Sharma HS, Hussain S, Schlager J, Ali SF, Sharma A (2010). Influence of nanoparticles on blood–brain barrier permeability and brain edema formation in rats. Acta Neurochir. Suppl..

[17] Fiore M, Sambri A, Zucchini R, Giannini C, Donati DM, De Paolis M (2021). Silver-coated megaprosthesis in prevention and treatment of peri-prosthetic infections: A systematic review and meta-analysis about efficacy and toxicity in primary and revision surgery. Eur. J. Orthop. Surg. Traumatol..

[18] Henderson ER, Groundland JS, Pala E, Dennis JA, Wooten R, Cheong D, Windhager R, Kotz RI, Mercuri M, Funovics PT (2011). Failure mode classification for tumor endoprostheses: Retrospective review of five institutions and a literature review. J. Bone Joint Surg. Am..

[19] Henderson ER, O'Connor MI, Ruggieri P, Windhager R, Funovics PT, Gibbons CL, Guo W, Hornicek FJ, Temple HT, Letson GD (2014). Classification of failure of limb salvage after reconstructive surgery for bone tumours: A modified system Including biological and expandable reconstructions. Bone Joint J..

[20] Springer BD (2015). The diagnosis of periprosthetic joint infection. J. Arthroplasty.

[21] Little RJA, Rubin DB (2002). Statistical Analysis with Missing Data.

[22] Shivaram A, Bose S, Bandyopadhyay A (2017). Understanding long-term silver release from surface modified porous titanium implants. Acta Biomater..

[23] Ward TT, Steigbigel RT (1978). Acidosis of synovial fluid correlates with synovial fluid leukocytosis. Am. J. Med..

[24] Scoccianti G, Frenos F, Beltrami G, Campanacci DA, Capanna R (2016). Levels of silver ions in body fluids and clinical results in silver-coated megaprostheses after tumour, trauma or failed arthroplasty. Injury.

[25] Hardes J, von Eiff C, Streitbuerger A, Balke M, Budny T, Henrichs MP, Hauschild G, Ahrens H (2010). Reduction of periprosthetic infection with silver-coated megaprostheses in patients with bone sarcoma. J. Surg. Oncol..

